# Disease of the Maxillary Antrum Successfully Treated through a Root Canal

**Published:** 1900-04

**Authors:** Cuvier R. Marshall

**Affiliations:** Detroit, Mich.


ARTICLE VI.

### DISEASE OF THE MAXILLARY ANTRUM SUCCESSFULLY TREATED THROUGH A ROOT CANAL.

CUVIER R. MARSHALL, A. M., M. D., DETROIT, MICH.

As professional advisers we hear so much of other people's adversities that our own little personal trials and afflictions are apt to be thrust entirely into the background. Yet it must be confessed that we all—to use the common vernacular—"have troubles of our own."

I trust I may be pardoned for presuming to relate a personal experience. The only plausible excuse I have to offer is a charitable desire to report the result of a simple mode of treatment for a condition which is not at all uncommon, and which is usually considered to be amenable to surgical intervention only.

Some months ago I became aware that I had an exposed pulp in the left upper first molar. A gold filling that had been placed there several years before had fallen out and the exposed cavity enlarged by caries until the pulp chamber was laid open. I consulted a dental practitioner, a young man of reputed ability, who stated that it would be necessary to devitalize the pulp, after the removal of which he could fill the root canals and pulp chamber. Accordingly that was done, as I was led to understand, and nothing more was thought of the matter. A short time afterward, while working hard and exposed much to inclement weather in responding to professional calls, I was seized with a severe rhinitis which I believed at the time had been contracted in the usual way. Notwithstanding the fact that the attack was accompanied with an unusually prefuse muco-purulent discharge of a very offensive nature, the real cause of the condition did not occur to me. Relief could be obtained only by the vigorous and almost constant use of antiseptic nasal sprays, of which a mixture of normal salt solution with one per cent. carbolic acid gave me the most satisfaction.

This state of affairs persisted for several weeks with alternate improvement and recrudescence. There was no pain or localized tenderness, nor, in fact, any other symptom to indicate the source of the trouble. Finally I became convinced, intuitively rather than rationally, that the site of the disease was the maxillary antrum, and that the molar tooth which had been filled was the exciting cause of my trouble. I then consulted a surgeon who made a careful examination, and while he could detect no objective evidence of antral disease he thought it would be well to have the suspected tooth extracted. I was somewhat reluctant to lose a serviceable tooth, and decided, upon my own responsibility, to have the filling removed if possible, in the hope that more light might thereby be thrown upon the etiology of the disease. With that object alone in view, I called upon a young dentist of considerable skill and good judgment, Dr. John Roche, of Detroit, to whom I related the circumstances, and who at once consented to make an effort to remove the amalgam filling. The drill was applied and had scarcely gotten in motion when it suddenly plunged through a mere shell of amalgam, with which the crown of the tooth had been capped giving exit to a small quantity of foul smelling pus. Further examination revealed the fact that one canal was freely open and communicated with the antrum, into which a slender probe could be readily passed, while the two remaining canals contained live pulp. The latter were cleared of their contents and filled, and appropriate treatment was begun in the open canal.

To my intense gratification the discharge from the nostril immediately ceased, but it would as promptly return whenever medicated cotton or any other impediment to free drainage was placed in the canal; indeed, the prospect of saving the tooth seemed to be as remote as ever. Realizing that a long course of treatment would be required to accomplish a cure, involving frequent applications of antiseptic solution, I therefore determined, with the acquiescene of my dental adviser, to treat the trouble for a time myself. This I did faithfully, with a result so eminently satisfactory that I am encouraged to go somewhat into detail in my narrative for the benefit of any one who may wish to profit by my experience.

I found that I could readily pass the needle of an ordinary hypodermatic syringe well up through the root canal, and by that means was enabled to thoroughly wash out the antrum, for with dilute hydrogen dioxide and next with an antiseptic solution. Perfect drainage fortunately existed through the nasal foramen, by which route all detritus came away freely. In a few days I discovered that it was no longer possible to force fluids into the antrum, as the sinus had evidently closed. After that occurred the nasal discharge never again reappeared.

The next step was to check the formation of pus at the apex of the diseased root. To that end various antiseptic solutions were persistently employed for several weeks, but as often as applied the peroxide test would reveal the presence of pus. At that juncture samples of euthymol and enformol, two excellent antiseptic preparations from the laboratories of Parke, Davis & Co., were plaeed in my hands. Euformol is of about the same composition as euthymol with the addition of five per cent. of formaldehyde.

I at once began using the euthymol, diluted with three parts of sterile water, for injecting the root canal, keeping the pulp chamber constantly packed with cotton saturated with euformol. As the latter contains formaldehyde, a gaseous germicide of considerable power, I thought this volatile substance would perhaps penetrate further than fluids into the apical pus cavity, and, permeating the entire canal, would tend to keep it aseptic. These applications were continued for several weeks, until at last the action of euthymol and euformol proved to be positively germicidal and therefore curative, for all evidence of the presence of pus had disappeared under their use.

The canal was now filled by Dr. Roche with a mixture of iodoform and some excipient, the nature of which I do not recall, and the pulp chamber was packed with cement. As several months have since elapsed, I feel well satisfied with the result, for I have escaped what might have proved a very serious condition, and moreover I have now a useful tooth.—*Items of Interest.*

